# Baseline serum metabolites predict fractures in individuals who were Black and had type 2 diabetes

**DOI:** 10.3389/fendo.2026.1777636

**Published:** 2026-06-02

**Authors:** Carolyn Chlebek, Valerie Bussberg, Niven R. Narain, Michael A. Kiebish, Yu-Hua Tseng, Clifford J. Rosen, Matthew D. Lynes

**Affiliations:** 1 Center for Molecular Medicine, MaineHealth Institute for Research, Scarborough, ME, United States; 2 BPGBio, Framingham, MA, United States; 3 Joslin Diabetes Center, Harvard Medical School, Boston, MA, United States; 4 Harvard Stem Cell Institute, Harvard University, Cambridge, MA, United States; 5 Graduate School of Biomedical Sciences and Engineering, University of Maine, Orono, ME, United States; 6 Tufts University School of Medicine, Tufts University, Boston, MA, United States

**Keywords:** biomarkers, ethnic studies, fracture risk, metabolomics, type 2 diabetes mellitus

## Abstract

**Background:**

Type 2 Diabetes Mellitus (T2D) is a metabolic disorder with increasing prevalence worldwide. Fractures are increased in people with T2D. Black patients have higher bone mineral density than White patients, suggesting the potential for distinct mechanisms for fracture within specific populations.

**Methods:**

To test whether changes to metabolism during T2D may contribute to skeletal fragility, we analyzed 465 targeted metabolites in serum collected at baseline from 571 participants (average age 62.1 years) in the Action to Control Cardiovascular Risk in Diabetes trial (ACCORD, ClinicalTrials.gov NCT00000620), a randomized clinical trial of patients with T2D. Participants with T2D were enrolled and serum was collected at baseline. These serum samples were obtained from the National Heart, Lung, and Blood Institute Biologic Specimen and Data Repository Information Coordinating Center. We focused exclusively on ACCORD participants who were Black, an understudied population with regards to fracture risk. Following enrollment, participants were randomized to intensive or standard glycemia strategies, which did not affect fracture or fall risk. Using the longitudinal data from ACCORD BONE, we compared the baseline serum metabolome of participants who later fractured with those who did not fracture.

**Results:**

Individual metabolite analysis revealed circulating metabolites that were significantly different at baseline in those that fractured versus those who did not. One metabolite had both adjusted and unadjusted p-values that reached significance: 7,8-dihydrofolate, which is involved in folate-dependent one-carbon metabolism, was greater in participants who later fractured compared to those who did not fracture. Importantly, several metabolites related to the tricarboxylic acid cycle were reduced in participants who later fractured, with significant unadjusted p-values.

**Conclusion:**

In summary, the metabolic differences identified here highlight the role of altered systemic metabolism and its relationship to fracture risk. Future investigations will determine if the identified metabolites serve as predictors of fracture in patients with T2D who are not Black.

## Introduction

Type 2 Diabetes Mellitus (T2D) is a metabolic disease affecting over 500 million people worldwide (1). The International Diabetes Federation projects that the global prevalence of T2D will rise to over 750 million people by 2045 (1). Rates of T2D diagnosis in the US vary by race, with disease prevalence in non-Hispanic Black Americans nearly doubling that in non-Hispanic White individuals (12.1% versus 6.9%). Compared to White Americans, Black individuals are also diagnosed with T2D earlier (2). Large clinical trials such as the Action to Control Cardiovascular Risk in Diabetes trial (ACCORD) (3) provide opportunities to examine risks associated with T2D in more specific patient populations, such as Black Americans.

T2D increases the risk of fracture (4). Both T2D (5) and fracture (6) are independently associated with increased mortality rates, which were additive in an analysis of Australian patients with T2D who fractured (7). Compared to White Americans, Black individuals have reduced incidence of fracture (8). Classic risk factors for fracture also vary between Black and non-Hispanic White individuals, including bone mineral density (9) and 25-hydroxyvitamin D levels (10). To identify mechanisms contributing to fracture risk within specific populations, separate analysis of participants by race is warranted (11, 12). Few studies have examined molecular mechanisms contributing fracture risk in Americans who are Black and have T2D (13). Clinical prediction of fracture risk in non-White populations is suboptimal (14), highlighting the need for race-specific predictors of fracture.

Changes in systemic metabolism during T2D, including hyperglycemia and insulin resistance, alter bone and increase fracture risk. Advanced glycation end-products accumulate during hyperglycemia and T2D, negatively affecting both mechanical properties of bone tissue (15) and osteogenic cellular activities (16). Insulin signaling positively regulates osteoblast differentiation (17), therefore changes in circulating insulin during diabetes negatively affects bone formation. High levels of systemic glucose in T2D patients may contribute to local changes in osteogenic function and cellular metabolism (18). Similarly, obesity and high body mass negatively affect bone quality and skeletal cell function (19).

As a metabolic disease, T2D induces systemic changes that may directly or indirectly affect the skeleton. Multiple organ systems are altered during metabolic disease, changing the molecular signature of various tissues. By examining the molecular changes within circulation, we can gain insight into systemic metabolic changes associated with future fracture risk. Mass spectrometry is an analytical tool used to identify specific small molecules known as metabolites. Changes to circulating metabolites occur in populations with T2D, and are unique in Black individuals (20). The use of circulating metabolites as a biomarker for T2D diagnosis and disease severity is an expanding area of research. We hypothesized that changes to circulating metabolites could be used to predict future fracture in individuals who were Black and had T2D.

The connection between systemic metabolic activity and fracture risk, particularly in individuals who are Black and have T2D, is underexplored. Changes to both local and systemic metabolism have long been associated with skeletal health (21). The correlation between global metabolite levels and musculoskeletal disease is still in its infancy, with most studies using bone mass to assess risk of osteoporotic fracture (22, 23). Therefore, we sought to identify potential metabolite predictors of fracture risk in a population of Black individuals with T2D, using data collected from ACCORD participants. In this exploratory analysis of a relatively small clinical dataset, to identify metabolites associated with future fracture, we identified significant differences using traditional significance cut-offs for both unadjusted and adjusted p-values (p < 0.05) as well as findings worthy of future investigation (unadjusted p < 0.05, adjusted p < 0.25).

## Materials and methods

### ACCORD trial

The ACCORD clinical trial was originally designed to investigate intensive versus standard glycemic control in Type 2 Diabetics at risk of cardiovascular disease (CVD) (3). Inclusion criteria for the ACCORD trial included participants diagnosed with T2D, with an HbA1c between 7.5-11%. Participants also were required to have either a history of or significant risk factors for CVD. Individuals with serious hypoglycemia complications were excluded, as well as those with body mass index (weight divided by the square of height) greater than 45 kg/m (2). Participants with ongoing medical therapy with known adverse interactions with glycemic interventions, such as corticosteroid or protease inhibitors, or those who experienced more than 10% body weight loss in the past six months were also excluded. Participant weight, height, waist circumference, BMI, baseline HbA1c, and fasting plasma glucose were recorded as previously reported (3). We performed Chi-Square tests to determine if sex or medication use was significantly different in individuals who later fractured.

### Identifying fractures in ACCORD data set

In this case-control study, data from ACCORD trials, including both baseline and follow-up physical exams, were obtained from the BioLINCC biological specimen and data repository. All procedures were performed in compliance with relevant laws and institutional guidelines. All data and biospecimens obtained for this study were de-identified and therefore this work was not considered research involving human subjects, as determined by the MaineHealth Research Compliance Office.

During the ACCORD trial, physical exams were performed at baseline and annually for up to seven years. In an ancillary study, ACCORD BONE determined that risk of fracture or falls was not associated with intensive glycemic control (24). During follow-up exams, participants were asked if they had broken or fractured a bone since their last annual visit. Doctors or health care providers then recorded whether participants had broken or fractured a bone since their previous ACCORD visit using the code “frac”. These reported fracture events were centrally adjudicated at the University of California, San Francisco, as previously described (24). 54 of the 77 ACCORD clinical sites assessed fractures as part of ACCORD BONE (24). For our analyses, we only included individuals who participated in ACCORD BONE and thus had records indicating whether or not a fracture had been sustained after baseline physical exams; participants with records associated with the ACCORD code “frac” were included for analysis. Any participant with a recorded fracture occurring after the baseline visit was considered to have sustained a fracture. All other participants had records of no fractures at their last visit of this trial, and served as our control group. Both nonvertebral and spine fractures were included in this analysis. Medication use, including osteoporosis drugs and thiazolidines, also was recorded at exams.

In summary, participants who were Black, had records as a part of ACCORD BONE, and for whom sufficient serum was available through BIOLINCC were included in the study. No other exclusion criteria was used.

### Metabolomic analysis

1,791 participants were Black and had records for “frac”, indicating their participation in ACCORD BONE. Of these 1,791 identified participants, sufficient sample was available in 571 (32%). Metabolites were analyzed in baseline serum samples that had been collected at the initial visit in these 571 participants.

To analyze metabolites in the serum samples, chromatography was performed using ultrahigh-performance liquid chromatography (UHPLC) as previously described (25). Metabolite extraction was achieved using a mixture of isopropanol, acetonitrile, and water at a ratio of 3:3:2 v/v. Ten microliters of each thawed serum sample were injected onto a ZIC-pHILIC column (EMD Millipore, Billerica, MA, USA) with dimensions of 150 x 4.6 mm, 5 µm. Metabolites were separated using an ACN/H2O with 20 mM ammonium carbonate (pH 9.2) gradient over a 29-minute period. A 10-minute re-equilibration time was carried out in between injections. We used the NEXERA XR UPLC system (Shimadzu, Columbia, MD, USA), coupled with the Triple Quad 5500 System (AB Sciex, Framingham, MA, USA) to perform hydrophilic interaction liquid chromatography analysis.

Detection was performed using an AB SCIEX 5500 QqQ mass spectrometer, operated in both negative and positive ion modes. Full scan MS data was collected from m/z 70–1000 and metabolites were identified in a targeted list of 465 metabolites. Real-time mass calibration was performed throughout the duration of sample analysis. Quality control was performed using a metabolite standards mixture and pooled samples applying the methodology previously described (26–29). A quality control sample containing a standard mixture of amino and organic acids purchased from Sigma-Aldrich as certified reference material, was injected daily to perform an analytical system suitability test, and monitor recorded signals day to day reproducibility as it was described (25, 30–32). A pooled quality control sample was obtained by taking an aliquot of the same volume of all samples from the study and injecting daily with a batch of analyzed samples to determine the optimal dilution of the batch samples and validate metabolite identification and peak integration. Collected raw data were manually inspected, merged, imputed, and normalized by the sample median. Metabolite identification was performed using in house authentic standards analysis. Raw metabolite abundances were used in all tests; the presence of metabolites with very low or nondetectable abundances in our dataset precluded log transformations of the data.

### Evaluating metabolotype

To identify sub-clusters of participants based on evaluation of all metabolites measured in the metabolomics panel, data was imported into R (RStudio, 2024.04.2 Build 764). Any metabolite that was not identified in the samples by mass spectrometry, thus resulting in a reading of zero, was removed prior to analysis. Principal component analysis was performed using R functions (prcomp). Within R, normalization during PCA was performed; data was scaled to unit variance and centered. To handle numerical instability, our R code utilized singular value decomposition. The components explaining the most variance across the entire dataset were examined.

For each principal component analysis, any clusters were first identified visually then further investigated with hierarchical clustering. To further examine visually-identified clusters, hierarchical clustering with Euclidean distances was performed using R functions (dist, hclust), minimizing cluster variance (ward.D2). The top 10 contributors to principal components of interest were identified and further examined. Similarly, feature importance scores were obtained to identify the top 10 metabolites contributing to hierarchical clustering.

### Identifying individual metabolite differences between fractured and non-fractured participants

All metabolites contained variance in both groups, and thus individual t-tests were performed in R to assess differences between participants who later fractured and those who had no recorded fractures. Adjusted p-values for t-tests were then calculated with the Benjamini-Hochberg False Discovery Rate using R functions (fuzzySim). In this exploratory analysis, metabolites with adjusted p-values less than 0.25 and unadjusted p-values less than 0.05 were considered worthy of further investigation and identified as interesting candidates for future analysis. To evaluate the predictive power of each of the identified metabolites, Receiver Operating Characteristic analysis was performed using R functions (pROC). Raw metabolite abundances were used to determine differences in individual metabolites between fractured and non-fractured participants.

### Identifying interactions between individual metabolites and baseline participant characteristics to predict fracture

To determine if the baseline characteristics of the participants affected the association of individual metabolites with fracture, we identified 18 potential covariates. The ability of baseline circulating metabolites to predict fracture was then evaluated both using a comprehensive model that included all variables as well as individual covariate interactions. Patient characteristics evaluated included (1) sex, (2) baseline age, (3) the number of years since diabetes diagnosis, (4) baseline glycosylated hemoglobin (HbA1c), (5) fasting plasma glucose, (6) neuropathy or nerve problems at baseline, (7) waist circumference, (8) body mass index (BMI), (9) baseline history of cardiovascular disease, (10) prescription of thiazolidinediones, (11) prescription of estrogen, (12) whether the participant had cigarettes smoked in the 30 days prior to study enrollment, (13) whether participant lives alone, (14) number of alcoholic drinks consumed weekly, (15) participant’s highest level of education, (16) estimated glomerular filtration rate (eGFR), (17) urinary albumin, and (18) of urinary albumin to creatinine ratio.

To control for all potential covariates simultaneously, we performed an ANCOVA to test the predictive power of each individual metabolite on fracture, given all patient covariates. Adjusted p-values were calculated using the Benjamini-Hochberg False Discovery Rate. In our exploratory research, adjusted p-values less than 0.25 and unadjusted p-values less than 0.05 were considered worthy of further investigation and identified as interesting candidates for future analysis.

We evaluated the predictive power of each metabolite (predictor) on fracture (outcome), given individual patient characteristics as covariates. A generalized linear model was performed separately for each patient characteristic. P-values for the interaction between metabolites and patient characteristics were adjusted by the total number of analyses using the Benjamini-Hochberg False Discovery Rate. Any metabolite with a notable interaction with any patient characteristic, defined in this exploratory analysis as unadjusted p-value less than 0.05 and adjusted p-value less than 0.25, was further evaluated by simple linear regression. Regressions were performed separately for participants who later fractured and those who had no recorded fractures. Notable simple linear regressions were considered those with a p-value less than 0.05 and r (2) greater than 0.15. The calibration slope and Brier score were calculated for notable generalized linear models to assess how well predictions matched events and overall accuracy, respectively.

## Results

### Participant characteristics

Of the 571 participants included in our metabolomics assessments, 7.0% experienced a fracture (**Table 1**). Female participants, representing 61% of the dataset, fractured at a higher rate than males (8.0% versus 5.4%), although this was not significantly different (p=0.23). Although most baseline characteristics were not statistically significant between individuals who fractured and those who did not fracture, the average number of alcoholic drinks consumed weekly was greater in participants who did not experience a fracture compared to those with record of fracture. No participant in this analysis received drugs associated with osteoporosis treatment throughout the duration of the study. 22% of participants included in our analysis were taking thiazolidinediones and were evenly distributed between those who fractured and those who did not fracture (p=0.97). Compared to participants with no record of fracture, the population of participants who later experienced a fracture also had similar proportions of estrogen therapy recipients (p=0.26), neuropathy or nerve problems (p=0.86), history of cardiovascular disease (p=0.98), cigarette smokers (p=0.74), and solitary residents (p=0.26). The 571 participants analyzed were distributed across the ACCORD treatment arms (**Supplementary Table 1**).

**Table 1:** Baseline characteristics of ACCORD participants whose samples were included for metabolomic analysis.

Characteristic	Total	No fractures	Fractured
n	571	531	40
Male	222	210	12
Female	349	321	28
Baseline Age (years)	62.1 (6.7)	62.1 (6.8)	62.7 (5.5)
Weight (kg)	93.2 (18.9)	93.3 (18.9)	91.8 (19.2)
Height (cm)	168.4 (9.3)	168.4 (9.4)	167.7 (8.1)
Waist Circumference (cm)	104.2 (16.3)	104.1 (16.4)	105.2 (14.6)
BMI (kg/m2)	32.8 (5.8)	32.8 (5.8)	32.6 (6.1)
Baseline HbA1c	8.58 (1.16)	8.58 (1.16)	8.64 (1.25)
Fasting Plasma Glucose (mg/dL)	166.9 (62.5)	166.4 (61.9)	173.0 (69.9)
Diabetes duration (years)	11.0 (7.7)	10.8 (7.6)	13.1 (8.7)
Neuropathy or Nerve Problems	135	126	9
History of Cardiovascular Disease	158	147	11
Cigarette Smokers	103	95	8
Number of Alcohol Drinks Consumed (weekly)*	0.5 (1.5)	0.5 (1.6)	0.2 (0.5)
Lives Alone	388	364	24
eGRP	94.5 (25.3)	94.5 (25.3)	94.2 (26.9)
Urinary Albumin	13.6 (34.3)	13.94 (34.4)	9.5 (32.4)
Urinary Albumin to Creatinine Ratio	127.2 (394.4)	126.2 (378.9)	140.6 (567.6)
Osteoporosis Drug Use	0	0	0
Estrogen Drug Use	34	30	4
TZD Drug Use	127	118	9

571 participants who participated in ACCORD BONE were included for additional metabolic profiling. All participants examined were Black and had T2D. Individuals who later fractured were separated from those with no recorded fractures. For all baseline characteristics, no differences were detected based on fracture status (p>0.05 for all baseline characteristics). Data for fractures was obtained from ACCORD BONE. * indicates p-value less than 0.05 by Student's t-test.

### Association of metabolotype and future fracture risk

**Figure 1:**
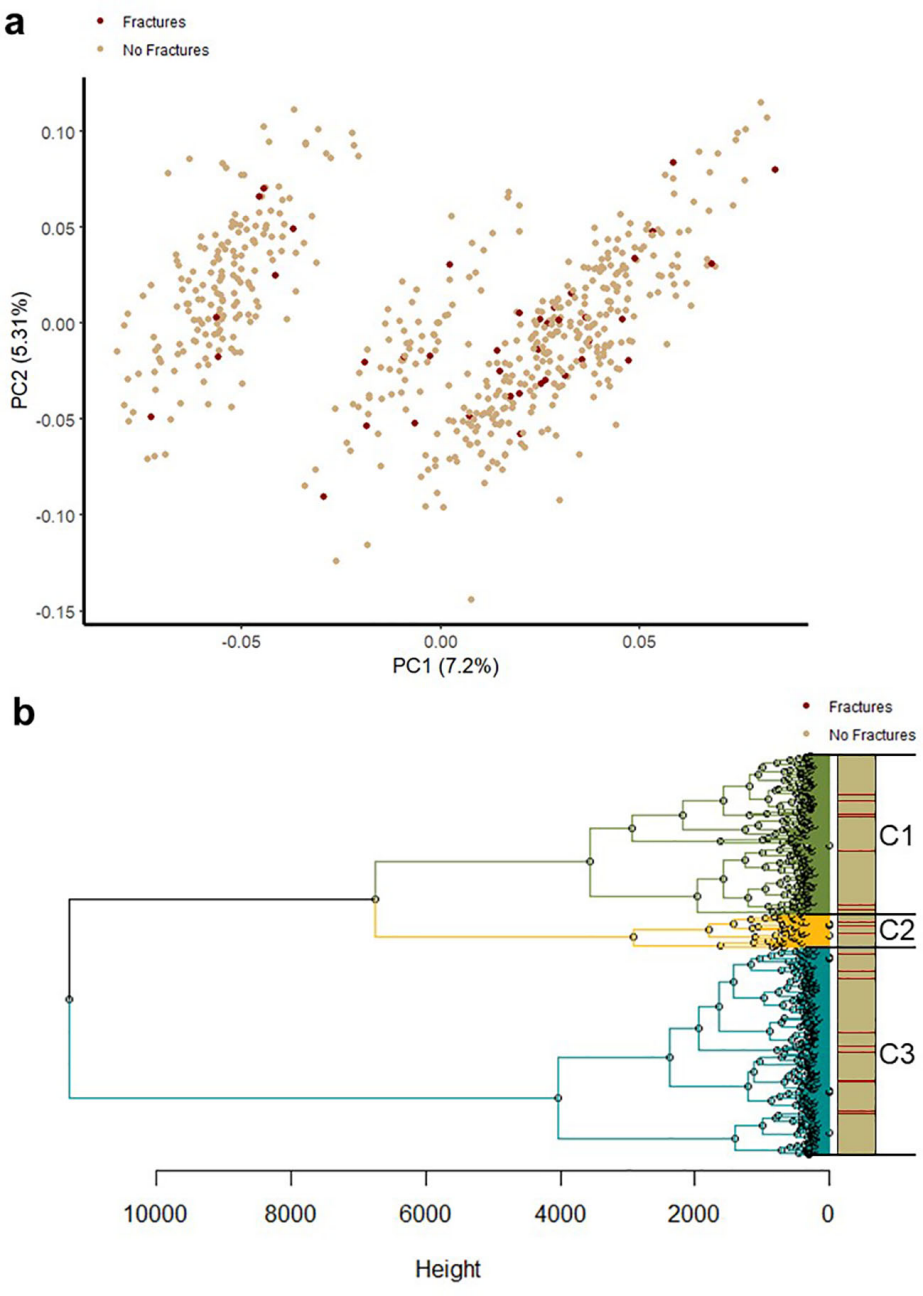
Baseline serum metabolomics from Black type 2 diabetic participants clustered into 3 metabolotypes. **(A)** Analysis of the circulating metabolites revealed three unique clusters that were evident in the principal component analysis, suggesting that ACCORD participants that were Black and had T2D also had unique metabolotypes. Participants later experiencing fractures (red dots) were distributed across all three clusters. **(B)** Hierarchical clustering of serum metabolites revealed that two clusters were more similar to each other than the third, indicated by cluster height. The three clusters are defined by color (green, yellow, teal) and by C1, C2, and C3. Fractured individuals were mapped within each cluster; the bar on the right of the dendrogram indicates fractures; participants with recorded fractures later in the study are indicated by red bars and non-fractured participants are indicated by brown. Fractured participants were distributed across all three hierarchical clusters.

To identify molecular mechanisms that may predict fracture, we performed mass spectrometry-based metabolomic profiling of baseline serum samples from these ACCORD participants. Unbiased clustering of all metabolites extracted from baseline serum was performed using principal component analysis. 385 principal components that explained more than 0% of variance were identified (**Supplementary Table 2**). Although not statistically different between fractured and nonfractured individuals, principal components 1 (PC1) and 2 (PC2) explained the greatest amount of variance within the dataset, 7.20% and 5.31%, respectively. Visual assessment of principal components 1 and 2 revealed three distinct clusters of participants, with fractured individuals present in all clusters (**Figure 1A**). Examining the top contributors to Principal Components 1 and 2, we identified that the top 10 metabolites included proline and cresol (**Table 2**). Carnitine, acetylcarnitine, betaine, choline, creatinine, glutamine, 1-palmitoyl-sn-glycero-3-phosphocholine, and histidine were also main drivers of principal component 1. Principal component 2 was similarly influenced by p-cresol sulfate, trimethylamine-N-oxide (TMAO), glucose, urea, dihydroxycholestanoyl taurine, nonanedioate, indoxyl sulfate, and 1-methyl histidine.

**Table 2:** Top metabolite contributors to principal components analysis and hierarchical clustering.

Metabolite	Contribution to PC1	Contribution to PC2	Dendrogram importance score
1-Methyl Histidine	0.038	0.083*	1.38
1-Palmitoyl-Sn-Glycero-3-Phosphocholine	0.159*	-0.068	8.83*
2-Methylglutaric Acid	0.013	-0.001	5.16*
Acetylcarnitine	0.363*	-0.085	20.38*
Betaine	0.472*	-0.096	27.32*
Carnitine	0.559*	-0.067	22.05*
Choline	0.175*	-0.089	6.08*
Creatinine	0.181*	0.042	11.68*
Cresol	0.129*	0.766*	1.72
Dihydroxycholestanoyl Taurine	0.029	0.078*	0.68
Glucose	0.002	0.093*	0.61
Glutamine	0.175*	-0.006	11.88*
Histidine	0.138*	-0.009	7.20*
Indoxyl Sulfate	0.007	0.055*	0.29
Nonanedioate	0.011	0.062*	0.34
P.Cresol Sulfate	0.026	0.419*	0.30
Proline	0.365*	0.058*	17.23*
TMAO	0.035	0.252*	0.37
Urea	0.091	0.078*	4.99

Asterisk following contribution value (*) indicates that metabolite was one of the top 10 contributors to that measure of interest.

To further evaluate the three apparent clusters visualized with PC1 and PC2, we performed hierarchical clustering with three clusters. Hierarchical clustering, displayed as a dendrogram, confirmed that two of the three clusters were more similar to each other than the third cluster (**Figure 1B**). Mirroring the principal component analysis of PC1 and PC2, participants who later experienced fractures were distributed across all three hierarchical clusters. Out of the top 10 metabolites driving the dendrogram clustering structure, 9 overlapped with those influencing the first principal component (**Table 2**). 2-Methylglutaric acid also impacted the dendrogram clustering structure.

Participants who later experienced fractures were distributed across all three clusters identified from metabolomic analyses. Clusters identified with PC1 and PC2 contained 7 or 26 participants who later experienced fractures whereas the hierarchical clusters within the 3-cluster dendrogram had 18, 3, or 19 participants. Notably, the strength of these clusters was low; the average silhouette score was 0.24. The largest cluster had modest clustering, indicated by a silhouette score of 0.33 whereas the smaller two clusters had scores of 0.14, suggesting weak clusters.

### Individual baseline circulating metabolites were associated with future fracture

Comparing participants who later fractured to those who had no recorded fractures, we found three metabolites of interest that had both p-values less than 0.05 and adjusted p-values less than 0.25 (**Table 3**). All three identified metabolites (4-guanidinocutanoate, NAD, and cortisol) were decreased in participants who later fractured, compared to those with no record of fracture. The identified metabolites had moderate sensitivity and specificity (**Table 3**).

**Table 3:** ACCORD fracture outcomes were associated with baseline metabolite levels.

Product	Non-fractured	Fractured	Unadjusted p-value	Adjusted p-value (FDR)	Effect size	95% confidence interval	Area under the curve	Sensitivity	Specificity
	*Average ± St.Dev*	*Average ± St.Dev*							
4-Guanidinobutanoate	0.7878 ± 0.6501	0.6093 ± 0.2518	0.0005	0.2099	-.01774	(-0.2743, -0.0805)	0.4593	0.6250	0.4331
NAD	0.0094 ± 0.0131	0.0060 ± 0.0051	0.0009	0.2099	-0.0034	(-0.0054, -0.0014)	0.5941	0.7750	0.4181
Cortisol	0.5114 ± 0.2067	0.4237 ± 0.1533	0.0014	0.2111	-0.0877	(-0.1396, -0.0358)	0.6246	0.5250	0.6780

At baseline, three metabolites were notably different between ACCORD participants who later fractured and those who did not fracture, as defined by having an unadjusted p-value less than 0.05 and an adjusted p-value less than 0.25. All metabolites had nonzero variance in both groups and were examined by a t-test (unadjusted p-values) followed by a multiple comparison correction (adjusted p-values). To evaluate the ability of each metabolite to predict fracture, receiver operating characteristic analysis was performed. The area under the curve, sensitivity, and specificity were evaluated.

### Covariate analysis considering participant characteristics known to affect fracture risk revealed additional baseline circulating metabolites that were associated with future fracture

We used ANCOVA analysis to evaluate the combined effects of each individual metabolite and all 18 covariates on risk of future fracture. In this model, four metabolites (4-pyridoxic acid, thiamine, 2,8-dihydroxyadenine, and ascorbic acid) had both adjusted and unadjusted p-values less than 0.05 (**Table 4**). We identified ten additional metabolites of interest, which had unadjusted p-values less than 0.05 and adjusted p-values less than 0.25.

**Table 4:** When models considered covariates previously associated with changes to the skeleton, the baseline abundances of ten metabolites were associated with fracture outcomes.

Product	Non-fractured	Fractured	Unadjusted p-value	Adjusted p-value (FDR)
	Average ± St.Dev	Average ± St.Dev		
4-Pyridoxic Acid	3.0782 ± 4.6913	10.9060 ± 24.4789	< 0.0001	< 0.0001
Thiamine	0.3268 ± 0.2686	0.5130 ± 0.5942	< 0.0001	0.0200
2,8-Dihydroxyadenine	0.1916 ± 0.7251	0.8366 ± 3.2300	0.0003	0.0337
Ascorbic Acid	0.0104 ± 0.0318	0.0387 ± 0.1400	0.0003	0.0337
Pyruvate	0.1431 ± 0.0420	0.1197 ± 0.0442	0.0006	0.0549
Cinnamoyl Glucoside	1.4706 ± 4.4857	7.5501 ± 40.0878	0.0013	0.0969
D-Glucarate	0.0150 ± 0.0080	0.0196 ± 0.0179	0.0017	0.1161
Folate	0.0014 ± 0.0057	0.0048 ± 0.0185	0.0026	0.1516
Furanedicarboxylcarnitine	0.8257 ± 1.0907	1.3834 ± 2.2635	0.0041	0.2131
Acetylhistamine	0.0857 ± 0.0690	0.1290 ± 0.2871	0.0050	0.2326

In this exploratory analysis, metabolites were considered notable with unadjusted p < 0.05 and adjusted p < 0.25 (False Discovery Rate multiple comparison correction). Four metabolites were significantly different between participants who later fractured and those who did not fracture (both adjusted and unadjusted p < 0.05). Using ANCOVA analysis, the predictive power of individual metabolites were evaluated with the consideration of 18 covariates.

### In participants who later experienced a fracture, baseline HbA1c and smoking history were significant covariates

**Figure 2:**
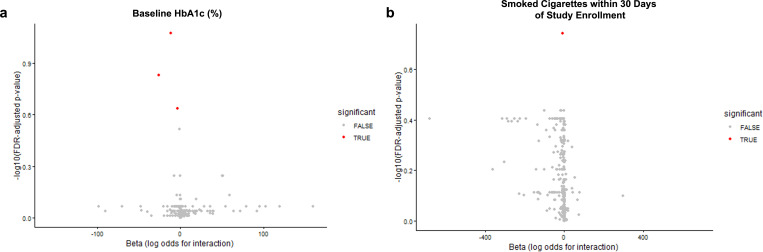
Individual metabolites were varied by fracture and patient characteristics. Following analysis with a generalized linear model, the notable interactions between **(A)** baseline HbA1c and **(B)** smoking status (determined based on whether a participant had smoked a cigarette within 30 days of study enrollment) were identified for individual metabolites.

**Figure 3:**
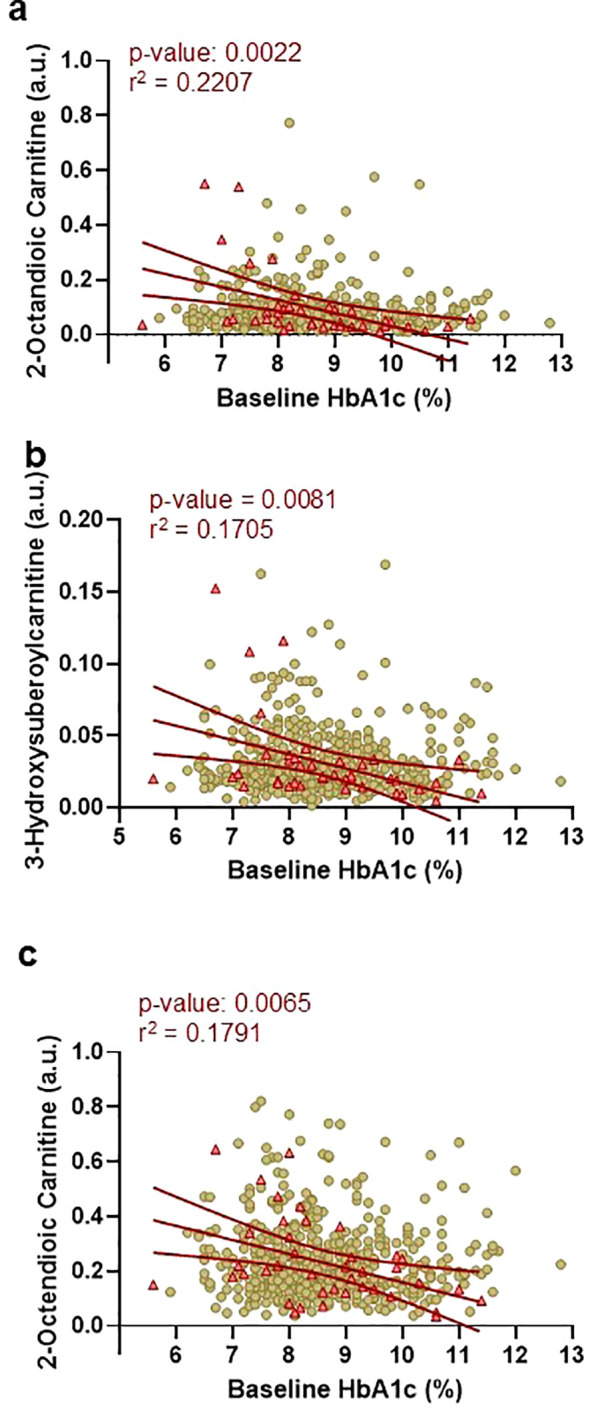
In participants who later experienced fracture, individual metabolites were negatively correlated with baseline HbA1c. In participants who later fractured, baseline HbA1c negatively correlated with 2-octandioic carnitine **(A)**, 3-hydroxysuberoylcarnitine **(B)**, 2-octendioic carnitine **(C)** were identified. No significant correlations between baseline HbA1c and individual metabolites were identified in participants who did not fracture (p > 0.05).

Using generalized linear models, interesting interactions between baseline HbA1c and whether a participant had smoked 30 days prior to study enrollment were identified for several individual metabolites (**Figure 2**). The calibration slope and Brier score of these models were calculated (**Supplementary Table 3**). For assessment of the continuous covariate (HbA1c), individual metabolites with notable interaction terms between fracture status and baseline HbA1c were further evaluated with simple linear regressions. Using a cutoff of p < 0.05, three significant correlations between baseline HbA1c and metabolite abundance were identified in participants who later experienced a fracture (**Figure 3**); the metabolite abundances of 2-octandioic carnitine, 3-hydroxysuberoylcarnitine, and 2-octendioic carnitine negatively correlated with baseline HbA1c in participants who later experienced a fracture. No significant correlations (p < 0.05) were identified in participants with no record of fracture.

In our analysis of participants who smoked within thirty days of enrollment, the interaction between adenine and smoking status was further investigated to predict fracture. In our model of active smokers (defined as having smoked a cigarette in the 30 days prior to enrollment), adenine concentration positively predicted fracture in smokers (slope of linear regression model= 3.81), but negatively predicted fracture in non-smokers (-3.65). Using a 95% confidence interval, we confirmed that these findings were statistically significant (p = 0.0004).

Additional covariates (sex, age, waist circumference, BMI, years since diabetes diagnosis, fasting plasma glucose, alcohol consumption, education level, living alone, neuropathy, prescriptions of thiazolidinediones or estrogen, history of cardiovascular disease, eGFR, urinary albumin, and albumin ratio) did not have notable individual interactions with any metabolites and thus lacked an effect on the predictability of fracture risk.

## Discussion

Using a large sample of individuals who were Black and had T2D, we identified individual serum metabolites that may serve as predictors of future fractures in this exploratory, hypothesis-generating analysis. We identified changes in metabolite abundances that should be further investigated with larger samples; notably, at baseline, the abundances of three metabolites (4-guanidinobutanoate, NAD, and cortisol) were lower in ACCORD participants who later experienced a fracture compared to those without fracture (based on adjusted p-values < 0.25). When baseline health characteristics that have been previously been associated with fracture were included in a covariate model, an additional 10 metabolites were identified as interesting candidates, 4 of which had both significant adjusted and unadjusted p-values. Finally, we identified interactions between baseline HbA1c and smoking status, highlighting the use of circulating metabolites in concert with health data as a potential future clinical application of this work.

T2D is associated with an increased risk of fracture (4), but there is significant controversy regarding major risk factors for fractures, particularly because bone mineral density is generally not diminished and in some cases is increased in T2D. Importantly, data on fracture risk among Black patients with impaired glucose intolerance and T2D is even more limited. But, despite lower rates of fracture in Black Americans compared to other races (8), in our dataset of 571 individuals, a sufficient number of participants fractured to allow us to identify potential predictors of bone health in T2D participants who were Black. Our fracture incidence of 7.5% was similar to previously reported rates of fracture in postmenopausal women who were Black (8).

Analysis of metabolite abundance in relation to health outcomes can both improve our understanding of the mechanisms of disease and also lay the groundwork for investigation of potential biomarkers. Given the metabolic nature of T2D, several studies have investigated the role of circulating metabolites for both prediction of insulin resistance as well as later complications. Levels of circulating metabolites related to the TCA cycle, such as pyruvate, have previously been linked to increased risk of developing T2D (33). Mitochondrial dysfunction is also evident within islet cells in models of T2D, and may contribute to the circulating metabolites measured here (34). Overall, the altered systemic homeostasis that is characteristic of T2D, including altered glucose, protein, and lipid metabolism, could have limited the clinical utility of our findings. The fact that all participants included in our study had T2D and thus altered circulating metabolite abundances could have limited our ability to detect significant, unadjusted metabolite abundances that predict fracture within this small cohort, further supporting our use of exploratory, hypothesis-generating analyses.

Racial differences in circulating metabolites have been identified in the context of T2D. Branched chain amino acids, including leucine, were identified as risk factors in Caucasian and Hispanic populations, but not in African American populations (20), highlighting the racial heterogeneity of T2D. Additional metabolomic investigations have identified unique metabolic profiles associated with development of T2D and obesity-related disease in participants who were Black or in geographical regions whose residents are primarily Black (35–38). Although metabolomics also has been employed to identify biomarkers associated with fracture risk, most studies have investigated metabolites in the context of osteoporosis and participants have been primarily White or Asian (22, 23). Furthermore, most of the studies evaluating fracture risk rely on measures of bone mass rather than future fracture records. Our investigation of the association between specific metabolites and fracture in the context of T2D for a cohort of Black participants is novel and addresses a critical patient population.

The metabolites identified in these analyses are hypothesis-generating and represent potential novel areas of investigation for understanding skeletal health. One metabolite related to amino acid metabolism, 4-guanidinobutanoate, was reduced at baseline in participants who later experienced a fracture (adjusted p-value <0.25) and currently has no defined roles in bone. Pyruvate and NAD (adjusted p-values <0.25) were both reduced in participants who later experienced fracture and are associated with the TCA cycle; in bone tissue, increases in glycolytic activity and decreases in the TCA cycle occur during aging (39), coincident with reductions in skeletal strength. Pyruvate, which also contributes to the TCA cycle, has been positively correlated with bone formation markers in humans (40). In mice, systemic inhibition of pyruvate-mediated oxidative phosphorylation reduced cortical bone mass (41). The reduced pyruvate measured here could reflect reductions in bone formation, due to lower substrate availability for the TCA cycle at baseline in the ACCORD participants. Similarly, NAD+ (42) is required for osteoblast function, and was reduced in individuals who fractured. Additionally, the effect of glucocorticoids on the skeleton have been previously examined (43); although increased cortisol levels are typically associated with low bone mass (44), our data demonstrated that participants who fractured had lower cortisol levels at baseline (adjusted p-value <0.25). In our study, lack of sample availability precluded us from measuring longitudinal changes to metabolite levels, so future studies evaluating fluctuations in cortisol and other metabolite levels prior to fracture may offer more insight into the role of these metabolites on bone health in patients with T2D.

In participants who later fractured, we identified correlations between baseline HbA1c or history of cigarette smoking with specific metabolites, some of which have established roles in fracture or T2D. In the absence of other health measures, only three metabolites were considered interesting and worthy of additional investigation regarding predicted fracture risk; however, when all covariates were considered, we identified an additional ten metabolites associated with future fracture. This finding highlights the importance of considering other patient characteristics to identify those at highest risk for fracture (14). Access to additional components of the US Fracture Risk Assessment Tool (US-FRAX), including bone mineral density and history of fracture or falls, could have further improved our model.

Although the metabolites identified in this study may serve as potential predictors of fracture, there are several limitations related to original study design as well as the participant health information and sample available through BioLINCC. The authors of this manuscript were not involved in the clinical trial design, ACCORD-BONE design, or the sample collection. First, we did not have longitudinal serum samples to confirm these metabolites were consistently altered with subsequent fractures. Other medical records associated with fracture, such as dual-energy x-ray absorptiometry imaging, history of patient falls, and US-FRAX components, was absent from our dataset. Our de-identified records of fracture lacked information on the fracture type and site, which may have introduced heterogeneity into our analysis. Future work should investigate the ability of circulating metabolites to predict fractures by site. Additionally, given that our records do not specify the exact time a fracture occurred following the collection of baseline serum, we were not able to evaluate fracture risk using survival models. In our relatively small clinical dataset, the 7.0% fracture rate in this population precluded us from separating our data into a training and testing set with regards to model generation, resulting in optimistic calibration slopes and Brier scores. To reduce the risks of overfitting, our generalized linear models did not evaluate all 465 metabolites simultaneously, but rather identified top candidate metabolites that were then evaluated individually or in concert with a small number of additional predictors. Importantly, all models and associations presented here should be further validated with testing data sets. Additionally, all ACCORD participants had impaired metabolic health at baseline, which likely negatively affected skeletal health; the lack of a nondiabetic control in the ACCORD study precluded us from comparing samples from participants with and without T2DM. Finally, the number of individuals who fractured in ACCORD is relatively small.

Our analysis of circulating metabolites and their association with future fracture had additional limitations. Additional metabolic differences between individuals who fractured versus nonfractured likely exist locally within bone cells and may not elicit changes to circulating factors. Cellular metabolism varies between bone and other tissues, such as in the bone marrow, and the relative contribution of these changes to the serum is not clear. Moreover, the individual metabolites identified here have limited prediction power, primarily due to low specificity. Given the low specificity of the identified metabolites, this data may be prone to Type I Error and thus should be considered as hypothesis-generating, with further validation of these results required. Further work should be done to understand if the metabolites identified here would extend to other racial groups or disease states.

This work is the first to identify specific metabolites that are worthy of future investigation, which regards to prediction of fracture in individuals who are Black and have T2D. Future work should validate the utility of these identified metabolites in other populations. Limited research has been conducted on minority groups, particularly with regards to fracture risk (8), adding to the novelty of this work. We identified more metabolites when considering relevant health characteristics as covariates, further emphasizing the need to consider changes in circulating metabolites in concert with participant health. In summary, our work underlines the systemic changes in individual metabolites that occur in T2D ACCORD participants prior to fracture.

## Data Availability

The datasets presented in this study can be found in online repositories. The names of the repository/repositories and accession number(s) can be found in the article/**Supplementary Material**.
