# Supplementary material for: Baseline serum metabolites predict fractures in individuals who were Black and had type 2 diabetes
Source: Front Endocrinol (Lausanne). 2026 Jun 2;17:1777636. doi: 10.3389/fendo.2026.1777636 (PMC13268917; doi:10.3389/fendo.2026.1777636)
Supplement: Supplementary file 1 [file Table1.docx]

**Table S1. Participants were distributed across the ACCORD treatment arms.**

| Treatment Arm | Participants (n) |
| --- | --- |
| Standard Glycemia/Standard BP | 71 |
| Standard Glycemia/Intensive BP | 100 |
| Standard Glycemia/Lipid Placebo | 58 |
| Standard Glycemia/Lipid Fibrate | 46 |
| Intensive Glycemia/Standard BP | 102 |
| Intensive Glycemia/Intensive BP | 86 |
| Intensive Glycemia/Lipid Placebo | 48 |
| Intensive Glycemia/Lipid Fibrate | 60 |

**Table S2. Principal component analysis of fractured versus nonfractured individuals.** The percent of variance explained by each principal component was calculated to measure the amount of total variability in the original data that a particular component captured. Within each principal component, a principal component score was calculated for each sample, then compared between fractured and nonfractured individuals using a t-test. 36 of the principal components had p-values less than 0.05, indicating that the principal component scores varied significantly between fractured and nonfractured individuals.

| **Principal Component** | **Statistical Testing of Principal Component Scores (p-value)** | **Percent Variance Explained (%)** |
| --- | --- | --- |
| PC1 | 0.0583 | 7.2 |
| PC2 | 0.5903 | 5.31 |
| PC3 | 0.9485 | 4.14 |
| PC4 | 0.7673 | 3.73 |
| PC5 | 0.1260 | 2.91 |
| PC6 | 0.7678 | 2.3 |
| PC7 | 0.6134 | 2.13 |
| PC8 | 0.0508 | 1.98 |
| PC9 | 0.1659 | 1.72 |
| PC10 | 0.5704 | 1.6 |
| PC11 | 0.0178 | 1.57 |
| PC12 | 0.2052 | 1.51 |
| PC13 | 0.5935 | 1.4 |
| PC14 | 0.0704 | 1.25 |
| PC15 | 0.1256 | 1.22 |
| PC16 | 0.8710 | 1.19 |
| PC17 | 0.5181 | 1.12 |
| PC18 | 0.7763 | 1.09 |
| PC19 | 0.2956 | 1.03 |
| PC20 | 0.7689 | 0.97 |
| PC21 | 0.8485 | 0.96 |
| PC22 | 0.4034 | 0.93 |
| PC23 | 0.0425 | 0.9 |
| PC24 | 0.4188 | 0.87 |
| PC25 | 0.2462 | 0.84 |
| PC26 | 0.3987 | 0.79 |
| PC27 | 0.8459 | 0.78 |
| PC28 | 0.5335 | 0.76 |
| PC29 | 0.3665 | 0.72 |
| PC30 | 0.3631 | 0.7 |
| PC31 | 0.0152 | 0.68 |
| PC32 | 0.0479 | 0.65 |
| PC33 | 0.1163 | 0.64 |
| PC34 | 0.0143 | 0.62 |
| PC35 | 0.4680 | 0.6 |
| PC36 | 0.0937 | 0.6 |
| PC37 | 0.0444 | 0.59 |
| PC38 | 0.1692 | 0.58 |
| PC39 | 0.5328 | 0.55 |
| PC40 | 0.3583 | 0.53 |
| PC41 | 0.1111 | 0.52 |
| PC42 | 0.1414 | 0.5 |
| PC43 | 0.2096 | 0.5 |
| PC44 | 0.0189 | 0.49 |
| PC45 | 0.0091 | 0.48 |
| PC46 | 0.5486 | 0.47 |
| PC47 | 0.0621 | 0.46 |
| PC48 | 0.5214 | 0.46 |
| PC49 | 0.8301 | 0.45 |
| PC50 | 0.9436 | 0.44 |
| PC51 | 0.1594 | 0.43 |
| PC52 | 0.0001 | 0.42 |
| PC53 | 0.0888 | 0.41 |
| PC54 | 0.0004 | 0.41 |
| PC55 | 0.0004 | 0.41 |
| PC56 | 0.6554 | 0.4 |
| PC57 | 0.6265 | 0.39 |
| PC58 | 0.0000 | 0.39 |
| PC59 | 0.9092 | 0.38 |
| PC60 | 0.0000 | 0.38 |
| PC61 | 0.4486 | 0.37 |
| PC62 | 0.1505 | 0.36 |
| PC63 | 0.0615 | 0.36 |
| PC64 | 0.0015 | 0.35 |
| PC65 | 0.0651 | 0.35 |
| PC66 | 0.0027 | 0.34 |
| PC67 | 0.5121 | 0.34 |
| PC68 | 0.8364 | 0.34 |
| PC69 | 0.4689 | 0.33 |
| PC70 | 0.7950 | 0.33 |
| PC71 | 0.8778 | 0.33 |
| PC72 | 0.4120 | 0.32 |
| PC73 | 0.0251 | 0.32 |
| PC74 | 0.3010 | 0.31 |
| PC75 | 0.0647 | 0.3 |
| PC76 | 0.0014 | 0.3 |
| PC77 | 0.8414 | 0.3 |
| PC78 | 0.1804 | 0.29 |
| PC79 | 0.9941 | 0.29 |
| PC80 | 0.2099 | 0.29 |
| PC81 | 0.0157 | 0.28 |
| PC82 | 0.8220 | 0.28 |
| PC83 | 0.0000 | 0.28 |
| PC84 | 0.1651 | 0.27 |
| PC85 | 0.0023 | 0.27 |
| PC86 | 0.0008 | 0.27 |
| PC87 | 0.5147 | 0.26 |
| PC88 | 0.5563 | 0.26 |
| PC89 | 0.0102 | 0.25 |
| PC90 | 0.3860 | 0.25 |
| PC91 | 0.0071 | 0.25 |
| PC92 | 0.7627 | 0.25 |
| PC93 | 0.1501 | 0.24 |
| PC94 | 0.1701 | 0.24 |
| PC95 | 0.8709 | 0.24 |
| PC96 | 0.4892 | 0.23 |
| PC97 | 0.8905 | 0.23 |
| PC98 | 0.0030 | 0.23 |
| PC99 | 0.6115 | 0.23 |
| PC100 | 0.9024 | 0.23 |
| PC101 | 0.0693 | 0.22 |
| PC102 | 0.6559 | 0.22 |
| PC103 | 0.0048 | 0.22 |
| PC104 | 0.6254 | 0.22 |
| PC105 | 0.0000 | 0.21 |
| PC106 | 0.3345 | 0.21 |
| PC107 | 0.3451 | 0.21 |
| PC108 | 0.0040 | 0.21 |
| PC109 | 0.0003 | 0.21 |
| PC110 | 0.3380 | 0.21 |
| PC111 | 0.7045 | 0.2 |
| PC112 | 0.1385 | 0.2 |
| PC113 | 0.0001 | 0.2 |
| PC114 | 0.1728 | 0.2 |
| PC115 | 0.0518 | 0.2 |
| PC116 | 0.0374 | 0.19 |
| PC117 | 0.5640 | 0.19 |
| PC118 | 0.4895 | 0.19 |
| PC119 | 0.7853 | 0.19 |
| PC120 | 0.0257 | 0.18 |
| PC121 | 0.0915 | 0.18 |
| PC122 | 0.5629 | 0.18 |
| PC123 | 0.0860 | 0.18 |
| PC124 | 0.5194 | 0.18 |
| PC125 | 0.5985 | 0.18 |
| PC126 | 0.4879 | 0.17 |
| PC127 | 0.6908 | 0.17 |
| PC128 | 0.0717 | 0.17 |
| PC129 | 0.4172 | 0.17 |
| PC130 | 0.6341 | 0.17 |
| PC131 | 0.5120 | 0.17 |
| PC132 | 0.7885 | 0.16 |
| PC133 | 0.3230 | 0.16 |
| PC134 | 0.2776 | 0.16 |
| PC135 | 0.8465 | 0.16 |
| PC136 | 0.1479 | 0.16 |
| PC137 | 0.9416 | 0.15 |
| PC138 | 0.6151 | 0.15 |
| PC139 | 0.4133 | 0.15 |
| PC140 | 0.2060 | 0.15 |
| PC141 | 0.0238 | 0.15 |
| PC142 | 0.1226 | 0.15 |
| PC143 | 0.6261 | 0.14 |
| PC144 | 0.8832 | 0.14 |
| PC145 | 0.1561 | 0.14 |
| PC146 | 0.4516 | 0.14 |
| PC147 | 0.0583 | 0.14 |
| PC148 | 0.0552 | 0.14 |
| PC149 | 0.4427 | 0.14 |
| PC150 | 0.2527 | 0.13 |
| PC151 | 0.1603 | 0.13 |
| PC152 | 0.3512 | 0.13 |
| PC153 | 0.5605 | 0.13 |
| PC154 | 0.0469 | 0.13 |
| PC155 | 0.9227 | 0.13 |
| PC156 | 0.0037 | 0.13 |
| PC157 | 0.7661 | 0.13 |
| PC158 | 0.3869 | 0.13 |
| PC159 | 0.1027 | 0.12 |
| PC160 | 0.2141 | 0.12 |
| PC161 | 0.8743 | 0.12 |
| PC162 | 0.3027 | 0.12 |
| PC163 | 0.9122 | 0.12 |
| PC164 | 0.0760 | 0.12 |
| PC165 | 0.8738 | 0.12 |
| PC166 | 0.0219 | 0.12 |
| PC167 | 0.1949 | 0.11 |
| PC168 | 0.0673 | 0.11 |
| PC169 | 0.2580 | 0.11 |
| PC170 | 0.0426 | 0.11 |
| PC171 | 0.3989 | 0.11 |
| PC172 | 0.9185 | 0.11 |
| PC173 | 0.6147 | 0.11 |
| PC174 | 0.4954 | 0.11 |
| PC175 | 0.7891 | 0.1 |
| PC176 | 0.5379 | 0.1 |
| PC177 | 0.3010 | 0.1 |
| PC178 | 0.5802 | 0.1 |
| PC179 | 0.4292 | 0.1 |
| PC180 | 0.5252 | 0.1 |
| PC181 | 0.8659 | 0.1 |
| PC182 | 0.6516 | 0.1 |
| PC183 | 0.5422 | 0.1 |
| PC184 | 0.7265 | 0.09 |
| PC185 | 0.4512 | 0.09 |
| PC186 | 0.5245 | 0.09 |
| PC187 | 0.4301 | 0.09 |
| PC188 | 0.9060 | 0.09 |
| PC189 | 0.5865 | 0.09 |
| PC190 | 0.7482 | 0.09 |
| PC191 | 0.4732 | 0.09 |
| PC192 | 0.4193 | 0.09 |
| PC193 | 0.9262 | 0.09 |
| PC194 | 0.4708 | 0.08 |
| PC195 | 0.3466 | 0.08 |
| PC196 | 0.1340 | 0.08 |
| PC197 | 0.8309 | 0.08 |
| PC198 | 0.4975 | 0.08 |
| PC199 | 0.5441 | 0.08 |
| PC200 | 0.9870 | 0.08 |
| PC201 | 0.7537 | 0.08 |
| PC202 | 0.5311 | 0.08 |
| PC203 | 0.9155 | 0.08 |
| PC204 | 0.6960 | 0.08 |
| PC205 | 0.8812 | 0.08 |
| PC206 | 0.4087 | 0.07 |
| PC207 | 0.8845 | 0.07 |
| PC208 | 0.6511 | 0.07 |
| PC209 | 0.8283 | 0.07 |
| PC210 | 0.5905 | 0.07 |
| PC211 | 0.9472 | 0.07 |
| PC212 | 0.1834 | 0.07 |
| PC213 | 0.6436 | 0.07 |
| PC214 | 0.8700 | 0.07 |
| PC215 | 0.6766 | 0.07 |
| PC216 | 0.7364 | 0.07 |
| PC217 | 0.3983 | 0.07 |
| PC218 | 0.8504 | 0.06 |
| PC219 | 0.6924 | 0.06 |
| PC220 | 0.5801 | 0.06 |
| PC221 | 0.2795 | 0.06 |
| PC222 | 0.7318 | 0.06 |
| PC223 | 0.4759 | 0.06 |
| PC224 | 0.7028 | 0.06 |
| PC225 | 0.3338 | 0.06 |
| PC226 | 0.9617 | 0.06 |
| PC227 | 0.9684 | 0.06 |
| PC228 | 0.8000 | 0.06 |
| PC229 | 0.9185 | 0.06 |
| PC230 | 0.5447 | 0.06 |
| PC231 | 0.5004 | 0.06 |
| PC232 | 0.4197 | 0.05 |
| PC233 | 0.6936 | 0.05 |
| PC234 | 0.7548 | 0.05 |
| PC235 | 0.9919 | 0.05 |
| PC236 | 0.6815 | 0.05 |
| PC237 | 0.8033 | 0.05 |
| PC238 | 0.4871 | 0.05 |
| PC239 | 0.9830 | 0.05 |
| PC240 | 0.6798 | 0.05 |
| PC241 | 0.8741 | 0.05 |
| PC242 | 0.9741 | 0.05 |
| PC243 | 0.8517 | 0.05 |
| PC244 | 0.7973 | 0.05 |
| PC245 | 0.7559 | 0.05 |
| PC246 | 0.8364 | 0.05 |
| PC247 | 0.3845 | 0.05 |
| PC248 | 0.5298 | 0.05 |
| PC249 | 0.9722 | 0.05 |
| PC250 | 0.6516 | 0.04 |
| PC251 | 0.8301 | 0.04 |
| PC252 | 0.7934 | 0.04 |
| PC253 | 0.8824 | 0.04 |
| PC254 | 0.7121 | 0.04 |
| PC255 | 0.8314 | 0.04 |
| PC256 | 0.9611 | 0.04 |
| PC257 | 0.9883 | 0.04 |
| PC258 | 0.6851 | 0.04 |
| PC259 | 0.7754 | 0.04 |
| PC260 | 0.8392 | 0.04 |
| PC261 | 0.7962 | 0.04 |
| PC262 | 0.7641 | 0.04 |
| PC263 | 0.7090 | 0.04 |
| PC264 | 0.9828 | 0.04 |
| PC265 | 0.8682 | 0.04 |
| PC266 | 0.8810 | 0.04 |
| PC267 | 0.9241 | 0.04 |
| PC268 | 0.8255 | 0.04 |
| PC269 | 0.5176 | 0.04 |
| PC270 | 0.9515 | 0.03 |
| PC271 | 0.9803 | 0.03 |
| PC272 | 0.8389 | 0.03 |
| PC273 | 0.9998 | 0.03 |
| PC274 | 0.6678 | 0.03 |
| PC275 | 0.9148 | 0.03 |
| PC276 | 0.9102 | 0.03 |
| PC277 | 0.8081 | 0.03 |
| PC278 | 0.6028 | 0.03 |
| PC279 | 0.9947 | 0.03 |
| PC280 | 0.6129 | 0.03 |
| PC281 | 0.7871 | 0.03 |
| PC282 | 0.6344 | 0.03 |
| PC283 | 0.9377 | 0.03 |
| PC284 | 0.9830 | 0.03 |
| PC285 | 0.9800 | 0.03 |
| PC286 | 0.8187 | 0.03 |
| PC287 | 0.9141 | 0.03 |
| PC288 | 0.8739 | 0.03 |
| PC289 | 0.8430 | 0.03 |
| PC290 | 0.8543 | 0.03 |
| PC291 | 0.9352 | 0.03 |
| PC292 | 0.8558 | 0.03 |
| PC293 | 0.8341 | 0.03 |
| PC294 | 0.9949 | 0.03 |
| PC295 | 0.8250 | 0.02 |
| PC296 | 0.9385 | 0.02 |
| PC297 | 0.8840 | 0.02 |
| PC298 | 0.9788 | 0.02 |
| PC299 | 0.6372 | 0.02 |
| PC300 | 0.8876 | 0.02 |
| PC301 | 0.9961 | 0.02 |
| PC302 | 0.6784 | 0.02 |
| PC303 | 0.9994 | 0.02 |
| PC304 | 0.8582 | 0.02 |
| PC305 | 0.7261 | 0.02 |
| PC306 | 0.9993 | 0.02 |
| PC307 | 0.9749 | 0.02 |
| PC308 | 0.7838 | 0.02 |
| PC309 | 0.8728 | 0.02 |
| PC310 | 0.9896 | 0.02 |
| PC311 | 0.7436 | 0.02 |
| PC312 | 0.9680 | 0.02 |
| PC313 | 0.7486 | 0.02 |
| PC314 | 0.9195 | 0.02 |
| PC315 | 0.7830 | 0.02 |
| PC316 | 0.9161 | 0.02 |
| PC317 | 0.8314 | 0.02 |
| PC318 | 0.9772 | 0.02 |
| PC319 | 0.7999 | 0.02 |
| PC320 | 0.8587 | 0.02 |
| PC321 | 0.8283 | 0.02 |
| PC322 | 0.7952 | 0.02 |
| PC323 | 0.8563 | 0.02 |
| PC324 | 0.9253 | 0.02 |
| PC325 | 0.8476 | 0.02 |
| PC326 | 0.9796 | 0.02 |
| PC327 | 0.8908 | 0.02 |
| PC328 | 0.9291 | 0.02 |
| PC329 | 0.9906 | 0.01 |
| PC330 | 0.8158 | 0.01 |
| PC331 | 0.8355 | 0.01 |
| PC332 | 0.9447 | 0.01 |
| PC333 | 0.9301 | 0.01 |
| PC334 | 0.8649 | 0.01 |
| PC335 | 0.8099 | 0.01 |
| PC336 | 0.8732 | 0.01 |
| PC337 | 0.9337 | 0.01 |
| PC338 | 0.8875 | 0.01 |
| PC339 | 0.9628 | 0.01 |
| PC340 | 0.8923 | 0.01 |
| PC341 | 0.8929 | 0.01 |
| PC342 | 0.9757 | 0.01 |
| PC343 | 0.9789 | 0.01 |
| PC344 | 0.9922 | 0.01 |
| PC345 | 0.9552 | 0.01 |
| PC346 | 0.9415 | 0.01 |
| PC347 | 0.9231 | 0.01 |
| PC348 | 0.9702 | 0.01 |
| PC349 | 0.9814 | 0.01 |
| PC350 | 0.9088 | 0.01 |
| PC351 | 0.9546 | 0.01 |
| PC352 | 0.7858 | 0.01 |
| PC353 | 0.9815 | 0.01 |
| PC354 | 0.9148 | 0.01 |
| PC355 | 0.9842 | 0.01 |
| PC356 | 0.9926 | 0.01 |
| PC357 | 0.9712 | 0.01 |
| PC358 | 0.8958 | 0.01 |
| PC359 | 0.9485 | 0.01 |
| PC360 | 0.9753 | 0.01 |
| PC361 | 0.9937 | 0.01 |
| PC362 | 0.9147 | 0.01 |
| PC363 | 0.9176 | 0.01 |
| PC364 | 0.9665 | 0.01 |
| PC365 | 0.9230 | 0.01 |
| PC366 | 0.8756 | 0.01 |
| PC367 | 0.9352 | 0.01 |
| PC368 | 0.9861 | 0.01 |
| PC369 | 0.9988 | 0.01 |
| PC370 | 0.9553 | 0.01 |
| PC371 | 0.9176 | 0.01 |
| PC372 | 0.9030 | 0.01 |
| PC373 | 0.9965 | 0.01 |
| PC374 | 0.8722 | 0.01 |
| PC375 | 0.9675 | 0.01 |
| PC376 | 0.9735 | 0.01 |
| PC377 | 0.9922 | 0.01 |
| PC378 | 0.9862 | 0.01 |
| PC379 | 0.9684 | 0.01 |
| PC380 | 0.9175 | 0.01 |
| PC381 | 0.9524 | 0.01 |
| PC382 | 0.9508 | 0.01 |
| PC383 | 0.8878 | 0.01 |
| PC384 | 0.9534 | 0.01 |
| PC385 | 0.9787 | 0.01 |

**Table S3. Calibration slope and Brier score for Generalized Linear Models of Covariates.**

| Generalized Linear Model | Calibration Slope | Brier Score |
| --- | --- | --- |
| (HbA1c)*(2-octandioic carnitine) | 1 | 0.0406 |
| (HbA1c)*(3-hydroxysuberoylcarnitine) | 1 | 0.0469 |
| (HbA1c)*(2-octendioic carnitine) | 1 | 0.0480 |
| (HbA1c)*(2-octendioic carnitine) | 1 | 0.0626 |
